# Transient receptor potential cation channel subfamily V member 1 expressing corneal sensory neurons can be subdivided into at least three subpopulations

**DOI:** 10.3389/fnana.2015.00071

**Published:** 2015-06-08

**Authors:** Abdulhakeem Alamri, Romke Bron, James A. Brock, Jason J. Ivanusic

**Affiliations:** Department of Anatomy and Neuroscience, University of MelbourneMelbourne, VIC, Australia

**Keywords:** TRPV1, polymodal nociceptor, cornea, primary afferent neurons, sensory neurons

## Abstract

The cornea is innervated by three main functional classes of sensory neurons: polymodal nociceptors, pure mechano-nociceptors and cold-sensing neurons. Here we explored transient receptor potential cation channel subfamily V member 1 (TRPV1) expression in guinea pig corneal sensory neurons, a widely used molecular marker of polymodal nociceptors. We used retrograde tracing to identify corneal afferent neurons in the trigeminal ganglion (TG) and double label *in situ* hybridization and/or immunohistochemistry to determine their molecular profile. In addition, we used immunohistochemistry to reveal the neurochemistry and structure of TRPV1 expressing nerve endings in the corneal epithelium. Approximately 45% of corneal afferent neurons expressed TRPV1, 28% expressed Piezo2 (a marker of putative pure mechano-nociceptors) and 8% expressed the transient receptor potential cation channel subfamily M member 8 (TRPM8; a marker of cold-sensing neurons). There was no co-expression of TRPV1 and Piezo2 in corneal afferent neurons, but 6% of TRPV1 neurons co-expressed TRPM8. The TRPV1 expressing corneal afferent neurons could be divided into three subpopulations on the basis of calcitonin gene-related peptide (CGRP) and/or or glial cell line-derived neurotrophic factor family receptor alpha3 (GFRα3) co-expression. In the corneal epithelium, the TRPV1 axons that co-expressed CGRP and GFRα3 ended as simple unbranched endings in the wing cell layer. In contrast, those that only co-expressed GFRα3 had ramifying endings that branched and terminated in the squamous cell layer, whereas those that only co-expressed CGRP had simple endings in the basal epithelium. This study shows that the majority of TRPV1 expressing corneal afferent neurons (>90%) are likely to be polymodal nociceptors. Furthermore, TRPV1 expressing corneal afferent neurons can be subdivided into specific subpopulations based on their molecular phenotype, nerve terminal morphology and distribution in the corneal epithelium.

## Introduction

Sensory neurons play an important role in protection of the cornea and in maintaining corneal epithelial integrity. There are three main functional phenotypes of sensory neurons that innervate the corneal epithelium: polymodal nociceptors, pure mechano-nociceptors and cold-sensing neurons (reviewed in Belmonte et al., 2004a,b). Of these, corneal polymodal nociceptors represent the largest population of corneal sensory neurons.

Corneal polymodal nociceptors respond to a number of different types of noxious stimuli, including mechanical, thermal and chemical. In cornea, polymodal nociceptors are the principal source of neuronal activity caused by chemical irritation and heat and, together with pure mechano-nociceptors, may contribute to pain that is evoked by mechanical stimuli (Belmonte and Giraldez, 1981; Tanelian and Beuerman, 1984; Belmonte et al., 1991). Corneal polymodal nociceptors are sensitive to a variety of endogenous chemical mediators that can be released by damaged corneal tissue, including protons, bradykinin, prostaglandins and other arachidonic acid metabolites (Belmonte et al., 1991, 1994; Chen et al., 1997a,b). They can also be sensitized (show decreased sensory threshold, enhanced responsiveness and spontaneous activity) by repeated noxious stimuli and by inflammatory mediators (Belmonte and Giraldez, 1981; Belmonte et al., 1991; Gallar et al., 1993). In addition, the neuropeptides substance P and calcitonin gene related peptide (CGRP) released from corneal polymodal nociceptors (Belmonte et al., 2003) have a trophic influence within the corneal epithelium (Reid et al., 1993). Together, these features contribute to a role for polymodal nociceptors in a number of pathologies related to the cornea, including allergic keratoconjunctivitis (Acosta et al., 2013), fibromyalgia (Gallar et al., 2009), herpes simplex virus keratitis (Gallar et al., 2010) and corneal sensory defects associated with diabetes mellitus (Neira-Zalentein et al., 2011). Corneal polymodal nociceptors have large receptive fields and conduction velocities consistent with their being C or Aδ fiber neurons (Belmonte and Giraldez, 1981; Belmonte et al., 1991).

The transient receptor potential cation channel subfamily V member 1 (TRPV1) is expressed by primary sensory neurons and is activated by capsaicin, low pH (pH 6) and noxious heat (>42°C; Caterina et al., 1997; Tominaga et al., 1998; Davis et al., 2000). TRPV1 knockout mice display altered responses to many of these stimuli, including noxious thermal stimulation, protons and capsaicin, and have reduced thermal hypersensitivity in the context of inflammation (Caterina et al., 2000). In addition, capsaicin exclusively activates polymodal nociceptors in the cornea (Belmonte et al., 1991) and capsaicin application to the human eye produces pain (Dupuy et al., 1988; Zollman et al., 2000). As a result of these findings, TRPV1 expression is proposed to be important for sensory transduction in polymodal nociceptors and has become a surrogate marker of polymodal nociceptors.

There are only a few documented studies of TRPV1 expression in corneal afferent neurons. In the rat trigeminal ganglion (TG), 37% of retrograde labeled corneal afferent neurons were reported to express TRPV1, and approximately three quarters of these also expressed CGRP (Murata and Masuko, 2006). However, cholera toxin subunit b was used as the retrograde tracer, and this is known to preferentially label sensory neurons with large cell bodies. Thus this proportion may not be determined relative to the total population of corneal afferent neurons. A lower proportion of TRPV1 expressing corneal afferent neurons (23%) in rat TG was reported in a subsequent study using a different tracer (Fluorogold) that labeled both small and larger neurons (Nakamura et al., 2007). Some TRPV1 expression has been reported in intra-epithelial fibers immuno-labeled in cryosections of the corneal epithelium (Guo et al., 1999; Murata and Masuko, 2006). The latter of these two studies showed that some TRPV1 expressing intra-epithelial fibers express substance P and/or CGRP, but did not explore their morphology. None of the above studies considered the identity of non-peptidergic TRPV1 expressing corneal afferent neurons (those that did not express substance P or CGRP), and so the extent to which TRPV1 expressing corneal afferent neurons can be subdivided on the basis of molecular phenotype remains to be established. Furthermore, the morphology of terminal endings of TRPV1 expressing corneal afferent has not been investigated.

The aims of the current study were to show that TRPV1 expressing corneal afferent neurons are distinct from cold-sensing neurons [expressing transient receptor potential cation channel subfamily M member 8 (TRPM8); Ivanusic et al., 2013; Bron et al., 2014] and pure mechano-nociceptors (expressing Piezo2; Bron et al., 2014), to determine the morphology of TRPV1 expressing nerve terminals in the corneal epithelium, and to define the molecular phenotype of TRPV1 expressing corneal afferent neurons and their nerve terminals.

## Methods and Materials

### Animals

A total of 25 tricolored guinea pigs (250–300 grams) were used in this study. All experiments conformed to the Australian National Health and Medical Research Council code of practice for the use of animals in research, and were approved by the University of Melbourne Animal Experimentation Ethics Committee.

### Retrograde Tracing, Tissue Preparation, *In Situ* Hybridization and Immunohistochemistry

Our protocols for retrograde tracing, tissue preparation (both TG sections and corneal whole-mounts), immunohistochemistry and *in situ* hybridization have been described in detail in previous publications (Ivanusic et al., 2013; Bron et al., 2014). They are described here briefly. To identify corneal afferent neurons, the retrograde tracer Fast Blue (FB) was applied to the cornea of guinea pigs anesthetized with ketamine (40 mg/kg; i.m.) and xylazine (5 mg/kg; i.m.). Following a 10 day survival period to allow for transport of the tracer to cell bodies in the TG, each animal was anesthetized with sodium pentabarbitone (80 mg/kg; i.p.), perfuse-fixed, and trigeminal ganglia isolated and prepared for cyro-sectioning. Multiple series of sections (1 in every 6 sections) were cut at 10 μm using a cryostat (−18°C) and collected on glass slides (Superfrost Plus, Menzel GmbH). In experiments in which we used *in situ* hybrization, FB fluorescence was imaged prior to performing *in situ* hybridization and later aligned with images taken after processing was completed.

Fam38B (Piezo2) and TRPM8 genes were identified in the guinea pig genome sequence and primers were designed as shown in Table 1. Antisense digoxigenin (DIG)-labeled cRNA for Piezo2 and fluorescein-labeled cRNA for TRPM8 were prepared as in Bron et al. (2014) and sections were hybridized in diluted probes overnight (at 60°C). TRPM8 was detected by overnight incubation (at 4°C) with an alkaline phosphatase (AP)-conjugated sheep-anti-fluorescein antibody (1:1500; Roche Diagnostics; Table 2) and a 30 min incubation in Fast Red substrate (Roche Diagnostics). Piezo2 was detected by overnight incubation (at 4°C) with an AP-conjugated sheep-anti-DIG antibody (1:2000; Roche Diagnostics; Table 2) and incubation with nitro blue tetrazolium chloride/5-bromo-4-chloro-3-indolyl phosphate substrate (NBT/BCIP; Roche #11681451001).

**Table 1:** **Primers used in this study**.

Gene	GenBank accession number	Forward primer	Reverse primer
Cavia Porcellus Piezo2 (Fam38B)	KF204566	GCTCGCCAGAGACCATGATCAAATG	TCTCTCTTGCCCTGGAAACACGGT
Cavia Porcellus TRPM8	KF204567	GCTCCACTCTGCTAACAAAAGCTC	GAATTCTAATACGACTCACTATAGGGAGACCAAGGTCTCGTTGTCCTCATTTT*
Cavia Porcellus TRPV1	AY513245.1	AGAAAAGAGCTTCCTGAAGTGCATG	GAATTCTAATACGACTCACTATAGGGAGACCAAAGGAAACAAGGAGAGGAAAG*

*The T7 promoter sequence is underlined.

**Table 2:** **Source and concentrations of the primary and secondary antisera used in this study**.

Primary antibody antigen	Immunogen	Manufacturer	Dilution used
Transient receptor potential channel vanilloid receptor 1 (TRPV1)	C-terminus of rat TRPV1 (824–838)	Alomone labs, Jerusalem, Israel; Rabbit polyclonal; # ACC030	1:1000
Calcitonin gene-related peptide (CGRP)	synthetic rat Tyr-CGRP (23–37)	Biogenesis, Bournemouth, UK; Goat polyclonal; #1720–9007	1:1000
Calcitonin gene-related peptide (CGRP)	synthetic rat CGRP	Sigma, Missouri, US; Rabbit polyclonal; #C8198	1:1000
Neurofilament 200 (NF200)	carboxyterminal tail segment of pig neurofilament H-subunit	Sigma; Missouri, US; Mouse monoclonal; #N0142	1:2000
B tubulin III	Microtubules from rat brain	Covance, Emeryville, CA (MMS-435P); Mouse monoclonal;	1:1000
Human neuronal protein (Hu)	Human HuD peptide (QAQRFRLDNLLN-C)	Molecular probes, Oregon, US; mouse monoclonal; #A21207	1:1000
**Secondary antibody**	**Manufacturer**		**Dilution used**
Donkey α Rabbit Alexa594	Molecular probes, Invitrogen; #A21207		1:200
Donkey α mouse Alexa647	Molecular probes, Invitrogen; #A31571		1:200
Donkey α mouse Alexa488	Molecular probes, Invitrogen; #A11001		1:200
Donkey α Goat Alexa488	Molecular probes, Invitrogen; #A11055		1:200
**cRNA detection**	**Manufacturer**		**Dilution used**
Sheep α digoxigenin (DIG) Fab’ fragments, alkaline phosphatase (AP) conjugated	Roche; #11093274910		1:2000
Sheep α fluorescein Fab’ fragments, alkaline phosphatase (AP) conjugated	Roche; #11426338910		1:1500

Immunohistochemistry was used to reveal expression of TRPV1, CGRP, glial cell line-derived neurotrophic factor family receptor alpha3 (GFRα3) and Neurofilament 200 (NF200) in retrograde labeled corneal afferent neurons. The source and concentration of antibodies used for immunohistochemistry is listed in Table 2. In the cases where immunohistochemistry was used in conjunction with *in situ* hybridization, hybridization was performed first and was followed immediately by immunohistochemistry.

Corneal whole-mount immunohistochemistry was performed on tissues from animals killed by stunning followed by decapitation. Eyes were enucleated and the corneas isolated and cut into quadrants and fixed in cold (4°C) Zamboni’s fixative (2% formaldehyde and 15% saturated picric acid) for 3 h. Each quadrant was processed free floating to reveal TRPV1, CGRP, GFRα3, and Class III β tubulin immuno-labeling through the full thickness, and across a wide area, of the cornea.

### Antibody Specificity and Characterization

Details of the specificity and characterization of the antibodies for CGRP, NF200, and Class III β tubulin and Anti-Hu have been provided in Bron et al. (2014) or Ivanusic et al. (2013). Here we provide details for antibodies we have not used previously.

Anti-TRPV1 (Alomone Labs, ACC-030) is a rabbit polyclonal antibody raised against the intracellular c-terminus of rat TRPV1 (824–838). Western blotting of TRPV1 transfected HEK 293 cells shows a band at ~100 kDa (manufacturer’s technical information). Immuno-labeling of TG neurons with this antibody is not present in TRPV1 knockout mice, but is present in wild-type controls (Everaerts et al., 2009). We have performed experiments to show that pre-adsorption with the manufacturer’s peptide (TRPV1 peptide control antigen for Cat #ACC030, Lot #ACC030AG0440, Alomone labs) at 0.1–1 μg/ml completely abolishes staining in guinea pig corneal epithelium and cryosections of rat dorsal root ganglion (DRG; data not shown). We have also confirmed complete colocalization of the TRPV1 antibody staining and mRNA expression (primers for *in situ* hybridization detailed in Table 1) in guinea pig TG neurons using double *in situ* hybridization and immunohistochemistry (data not shown). Anti-GFRα3 (R + D Systems, AF2645) is a goat polyclonal antibody raised against the purified recombinant mouse GFRα3 extracellular domain (Glu34-Arg379). Using direct ELISA, this antibody shows less than 2% cross-reactivity with recombinant mouse GFRα2 or GFRα4, or recombinant rat GFRα1 (manufacturer’s information). Immuno-labeling of DRG neurons with this antibody is not present in GFRα3 knockout mice, but is present in wild-type controls (Fasanella et al., 2008). The specificity of secondary antibodies was tested with omission of the primary antibodies, which always resulted in no immunostaining.

### Image Acquisition and Data Analysis

To determine the proportion of retrograde labeled corneal afferent neurons that expressed TRPV1, Piezo2 and/or TRPM8, images of sections throughout the entire TG were captured at 20× using a VSlide Fluorescent Slide Scanner (MetaSystems, Carl Zeiss Pty, North Ryde BC NSW, Australia) and stitched together using the Metafer platform. Brightfield images of *in situ* hybridization involving NBT/BCIP staining (Piezo2) were inverted to facilitate comparison with images of immunofluorescence and Fast Red fluorescence *in situ* hybridization staining (TRPM8). To determine the proportion of retrograde labeled corneal afferent neurons that expressed TRPV1/CGRP and/or NF200, TRPV1/GFRα3 and/or NF200, and CGRP/GFRα3 and/or NF200, sections were examined and photographed using 10× or 20× objective magnification on a Zeiss Axioskope Z1 fluorescence microscope (Zeiss, Oberkocken, Germany). Only neurons with a nucleus visible in at least one channel were counted. Hu immuno-labeling was used to identify nucleated neurons that were not labeled with any of the other markers. Proportions were determined for each animal and are expressed as mean ± standard deviation. Size measurements (cross-sectional area of soma) for corneal afferent neurons were made using Zen Lite 2011 (Zeiss, Oberkocken, Germany). We classified corneal afferent neurons on the basis of size and myelination (NF200-immunoreactive) status as small (small unmyelinated, <800 μm^2^), medium (small myelinated, 800–1800 μm^2^) or large [large myelinated, >1800 μm^2^; see Bron et al. (2014) for a full description of this classification scheme].

Nerve fibers and terminals in the corneal epithelium were imaged using confocal microscopy (a META LSM510 or a Pascal confocal microscope with Zen imaging software, v5.5, Carl Zeiss MicroImaging, GmbH). Z-series of optical slices were collected to examine the distribution of nerve fibers and terminals through the full thickness of the corneal epithelium at multiple magnifications (20× and 40× objectives). For each objective, the pinhole was set to one Airy unit so that optimal slice thickness was obtained for that magnification. Figures were prepared using Imaris and CorelDraw software. Individual images were contrast and brightness adjusted and pseudo-colored when required to illustrate co-expression. No other manipulations were made to the images.

## Results

### The Majority of TRPV1-IR Corneal Afferent Neurons are Distinct from those that Express TRPM8 or Piezo2

**Figure 1:**
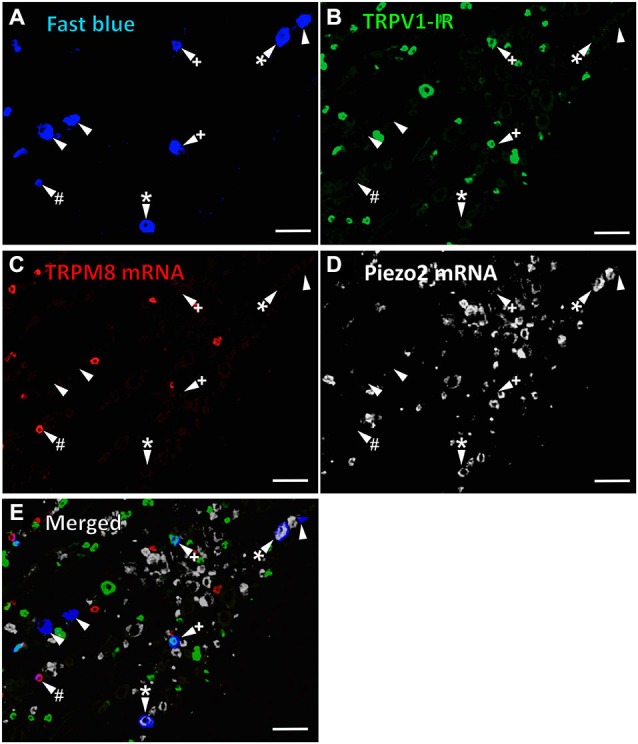
**TRPV1-IR, TRPM8 and Piezo2 expression in retrogradely labeled corneal afferent neurons**. The images in all panels are of the same field of a single section through the ophthalmic division of the trigeminal ganglion. **(A)** Retrograde labeled corneal afferent neurons (blue) imaged through a DAPI filter. **(B)** TRPV1-IR (green) imaged through a FITC filter. **(C)** TRPM8 expression (red) imaged through a TRITC filter. **(D)** Piezo2 expression (white) imaged using brightfield and inverted for ease of visualization. **(E)** Merged. Arrowheads highlight the same corneal afferent neurons throughout. Plus signs (+) highlight TRPV1-IR corneal afferent neurons. Hashes (#) highlight TRPM8 expressing corneal afferent neurons. Asterisks (*) highlight corneal afferent neurons that express Piezo2. Scale bars = 100 μm.

**Figure 2:**
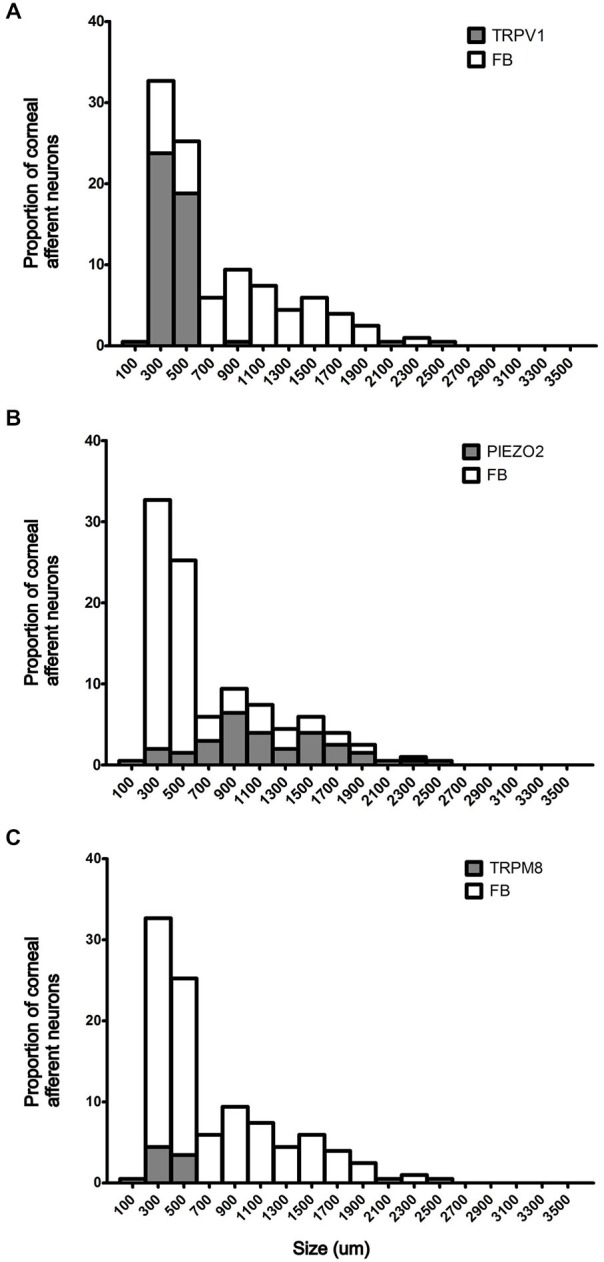
**Size/frequency distributions of subpopulations of Fast Blue (FB) labeled corneal afferent neurons identified in this study. (A)** TRPV1 expressing corneal afferent neurons (gray) were small. **(B)** Piezo2 expressing corneal afferent neurons (gray) were medium to large sized. **(C)** TRPM8 expressing corneal afferent neurons (gray) were small.

In the first series of experiments, we used retrograde tracing, *in situ* hybridization and immunohistochemistry to investigate the expression of TRPV1, TRPM8, and Piezo2 in retrogradely labeled corneal afferent neurons (Figure 1). We found 43% of the retrograde labeled corneal afferent neurons were TRPV1-IR, 8% expressed TRPM8 mRNA, and 28% expressed Piezo2 mRNA (*n* = 3; Table 3). There was no co-expression of TRPV1 with Piezo2 in corneal afferent neurons, and TRPV1 and TRPM8 were co-expressed in only a very small proportion of corneal afferent neurons (2%; Table 3). 31% of TRPM8 expressing corneal afferent neurons were TRPV1-IR and 6% of TRPV1-IR corneal afferent neurons expressed TRPM8 (Table 3). TRPV1-IR corneal afferent neurons were small and could be distinguished from Piezo2, but not TRPM8 expressing neurons, on the basis of size (Figure 2).

**Table 3:** **TRPV1-IR, TRPM8, and Piezo2 expression in corneal afferent neurons (experimental series 1)**.

Animal	Number of corneal afferent neurons	%TRPV1+ corneal afferent neurons	%Piezo2+ corneal afferent neurons	%TRPM8+ corneal afferent neurons	%TRPV1+ corneal afferent neurons that express TRPM8	%TRPM8+ corneal afferent neurons that express TRPV1
#1	75	48	27	9	6	29
#2	64	42	30	8	7	40
#3	63	40	27	6	4	25
Mean ± SD		43 ± 4	28 ± 2	8 ± 1	6 ± 1.7	31 ± 7.8

### Subpopulations of TRPV1-IR Corneal Afferent Neurons Could be Distinguished on the Basis of their Molecular Phenotype

**Figure 3:**
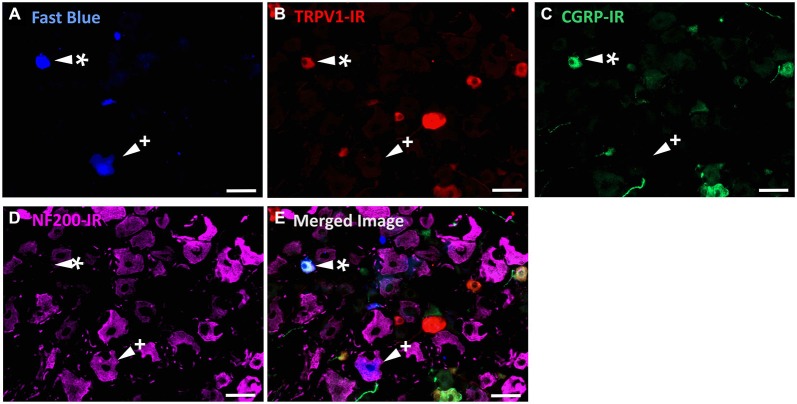
**TRPV1-IR, CGRP-IR, and NF200-IR in corneal afferent neurons**. The images in all panels are of the same field of a single section through the ophthalmic division of the trigeminal ganglion. **(A)** Retrograde labeled corneal afferent neurons (blue) imaged through a DAPI filter. **(B)** TRPV1-IR (red) imaged through a TRITC filter. **(C)** CGRP-IR (green) imaged through a FITC filter. **(D)** NF200-IR (magenta) imaged through a Cy5 filter. **(E)** Merged. Arrowheads highlight the same corneal afferent neurons throughout. Asterisk (*) highlights a corneal afferent neuron that expresses TRPV1 and CGRP but not NF200. Plus sign (+) highlights a corneal afferent neuron that expresses NF200 but not TRPV1 or CGRP. Scale bars = 50 μm.

**Figure 4:**
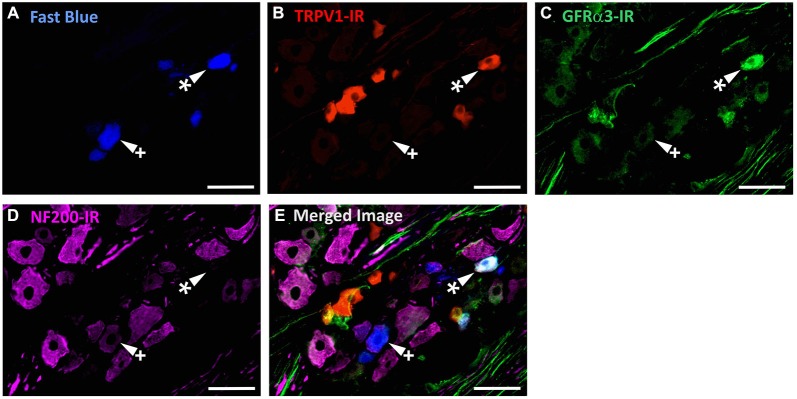
**TRPV1-IR, GFRα3-IR, and NF200-IR in corneal afferent neurons**. The images in all panels are of the same field of a single section through the ophthalmic division of the trigeminal ganglion. **(A)** Retrograde labeled corneal afferent neurons (blue) imaged through a DAPI filter. **(B)** TRPV1-IR (red) imaged through a TRITC filter. **(C)** GFRα3-IR (green) imaged through a FITC filter. **(D)** NF200-IR (magenta) imaged through a Cy5 filter. **(E)** Merged. Arrowheads highlight the same corneal afferent neurons throughout. Asterisk (*) highlights a corneal afferent neuron that expresses TRPV1 and GFRα3 but not NF200. Plus sign (+) highlights a corneal afferent neuron that expresses NF200 but not TRPV1 or GFRα3. Scale bars = 50 μm.

**Figure 5:**
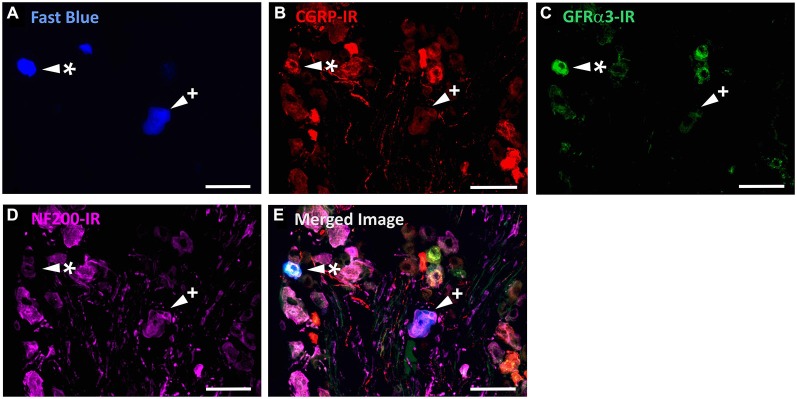
**CGRP-IR, GFRα3-IR, and NF200-IR in corneal afferent neurons**. The images in all panels are of the same field of a single section through the ophthalmic division of the trigeminal ganglion. **(A)** Retrograde labeled corneal afferent neurons (blue) imaged through a DAPI filter. **(B)** CGRP-IR (red) imaged through a TRITC filter. **(C)** GFRα3-IR (green) imaged through a FITC filter. **(D)** NF200-IR (magenta) imaged through a Cy5 filter. **(E)** Merged. Arrowheads highlight the same corneal afferent neurons throughout. Asterisk (*) highlights a corneal afferent neuron that expresses CGRP and GFRα3 but not NF200. Plus sign (+) highlights a corneal afferent neuron that expresses NF200 but not CGRP or GFRα3. Scale bars = 50 μm.

In the second series of experiments, we used retrograde tracing and immunohistochemistry to investigate the possibility that TRPV1-IR corneal afferent neurons could be further subdivided into distinct subpopulations on the basis of their molecular phenotype. We focused on those that are peptidergic nociceptors (CGRP-IR) and/or GFRα3–IR because CGRP is released in response to corneal application of heat or chemical irritants (such as capsaicin), but not cold, suggesting that some polymodal nociceptors contain CGRP (Belmonte et al., 2003), and because GFRα3 activation contributes to sensitization of capsaicin-sensitive (TRPV1+) DRG neurons (Malin et al., 2006). Due to lack of available and appropriate antibodies directed against TRPV1, CGRP, and GFRα3 (they were not raised in different species), we were unable to carry out triple labeling experiments for all three markers. However, we were able to perform multiple double label experiments using antibodies to two of these markers together with anti-NF200 (Figures 3–5) to define three separate populations of TRPV1-IR corneal afferent neurons. Half of the TRPV-IR corneal afferent neurons we counted were CGRP-IR (Table 4) and half were GFRα3–IR (Table 4). Almost all CGRP-IR and all GFRα3-IR corneal afferent neurons expressed TRPV1 (Table 4). Approximately half of all GFRα3-IR neurons expressed CGRP, but many did not (Table 5), and approximately half of all CGRP-IR neurons expressed GFRα3-IR, but many did not (Table 5). Few neurons in any of these subpopulations expressed NF200 (Table 5).

**Table 4:** **Molecular phenotype of TRPV1-IR corneal afferent neurons (experimental series 2)**.

Double label immunohistochemistry to identify TRPV1-IR and CGRP-IR							
Animal	Number of corneal afferent neurons	%TRPV1+ corneal afferent neurons	%CGRP+ corneal afferent neurons	%TRPV1+ corneal afferent neurons that are CGRP+	%CGRP+ corneal afferent neurons that are TRPV1+	%TRPV1+ corneal afferent neurons that are NF200+	%TRPV1+/CGRP+ corneal afferent neurons that are NF200+
#1	174	40	29	62	84	4	3
#2	293	39	22	47	83	12	2
#3	166	50	25	46	90	17	4
#4	215	42	23	46	80	6	1
Mean ± SD		42 ± 5	25 ± 3	50 ± 8	84 ± 4	10 ± 6	3 ± 0.01
**Double label immunohistochemistry to identify TRPV1-IR and GFRα3-IR**							
**Animal**	**Number of corneal afferent neurons**	**%TRPV1+ corneal afferent neurons**	**%GFRα3+ corneal afferent neurons**	**%TRPV1+ corneal afferent neurons that are GFRα3+**	**%GFRα3+ corneal afferent neurons that are TRPV1+**	**%TRPV1+ corneal afferent neurons that are NF200+**	**%TRPV1+/GFRα3+ corneal afferent neurons that are NF200+**
#1	143	34	24	51	71	16	5
#2	204	42	23	48	89	8	3
#3	148	47	28	57	93	10	8
#4	227	38	25	54	82	5	4
Mean ± SD		40 ± 6	25 ± 2	53 ± 4	84 ± 10	10 ± 5	5 ± 0.02

**Table 5:** **CGRP-IR, GFRα3-IR, and NF200-IR in corneal afferent neurons (experimental series 2)**.

**Double label immunohistochemistry to identify CGRP-IR and GFRα3-IR**							
Animal	Number of corneal afferent neurons	%CGRP+ corneal afferent neurons	%GFRα3+ corneal afferent neurons	%CGRP+ corneal afferent neurons that are GFRα3+	%GFRα3+ corneal afferent neurons that are CGRP+	%CGRP+ corneal afferent neurons that are not GFRα3+	%GFRα3+ corneal afferent neurons that are not CGRP+
#1	152	25	22	47	55	53	45
#2	288	24	29	52	43	48	57
#3	168	25	29	62	54	38	46
#4	202	26	26	43	44	57	56
Mean ± SD		25 ± 1	27 ± 3	51 ± 8	49 ± 6	49 ± 8	51 ± 6
**NF200 immuno-labeling**							
**Animal**	**Number of corneal afferent**	**%NF200+**	**%CGRP+/GFRα3- that are NF200+**		**%GFRα3+/CGRP- that are NF200+**		**%CGRP+/GFRα3+ that are NF200+**
#1	152	55	10		60		28
#2	288	43	9		27		11
#3	168	40	19		19		0
#4	202	35	3		14		0
Mean ± SD		43 ± 8	10 ± 7		30 ± 21		10 ± 13

### TRPV1-IR Intra-Epithelial Nerve Terminals Could be Distinguished on the Basis of their Morphology and Molecular Phenotype

In the third series of experiments, we explored the morphology and molecular phenotype of TRPV1-IR nerve terminals in whole-mounts of corneal epithelium. Double labeling with a pan–neuronal marker (mouse β tubulin-IR) allowed comparison with the morphology of non-TRPV1-IR nerve terminals. Co-labeling for CGRP-IR and/or GFRα3-IR was used, as above, to determine if TRPV1-IR nerve terminals could be subdivided on the basis of their molecular phenotype. In these experiments, we used high-resolution confocal microscopy to investigate the morphology and neurochemistry of TRPV1-IR nerve terminals in whole-mounts of corneal epithelium.

**Figure 6:**
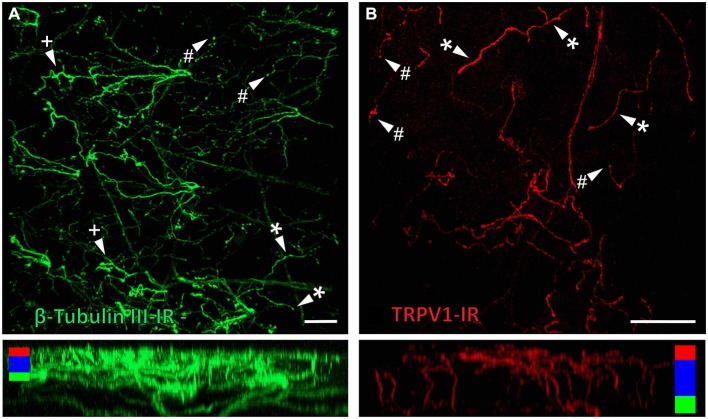
**Confocal images of nerve terminals in two different corneal whole-mount preparations labeled with antibodies directed against β tubulin III (A) and TRPV1 (B)**. Images are projected from *z*-series through the corneal epithelium. The small panels under **(A)** and **(B)** show orthogonal views generated by projecting the *z*-series above it in the *x*-plane. Arrowheads in **(A)** and **(B)** point to simple (#) nerve terminals in the wing cell layers and ramifying (*) or complex (+) nerve terminals in the squamous cell layer. Colored bars in the orthogonal projections indicate the approximate locations of the basal epithelium (green), wing cell layers (blue) and the squamous cell layer (red). Scale bar in *A* = 100 μm. Scale bar in *B* = 50 μm.

β-Tubulin III-IR nerve fibers were clearly identified in large nerve bundles in the corneal stroma. Nerves branched from these bundles and extended into the sub-epithelial plexus below the corneal epithelium, before they penetrated through Bowman’s membrane to enter the basal layer of the epithelium. In the basal layer they formed a complex network of leash fibers that ran parallel to the surface of the cornea. Some nerve fibers leave the basal layer and project up towards the surface of corneal epithelium. Their terminations can be described as simple, ramifying and complex endings. Simple terminals end in the wing cell layers with small single bulbar endings (Figure 6A). Ramifying nerve terminals project to the most superficial layer of the epithelium where they branch into three or four fibers that each run parallel to the surface and terminate with a single bulbar ending (Figure 6A). Complex terminals branch extensively as they ascend through the wing cell layers and end with multiple, large bulbar endings in the most superficial layer of the epithelium (Figure 6A). There were also some simple nerve terminals ending in the basal epithelium.

**Figure 7:**
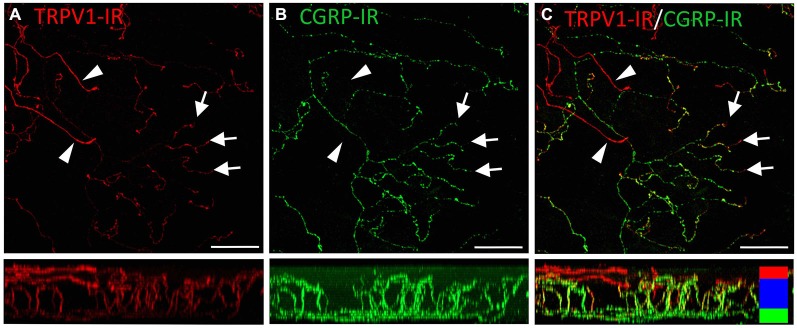
**Confocal images of TRPV1-IR (A) and CGRP-IR (B) nerve terminals in the same field of view in the corneal epithelium**. Images are projected from *z*-series through the entire corneal epithelium. The small panels under each image show orthogonal views generated by projecting the *z*-series above it in the *x*-plane. Arrowheads point to TRPV1-IR ramifying nerve terminals in the squamous cell layer and arrows point to simple endings in the wing cell layer that are both TRPV1- and CGRP-IR. The TRPV1-IR and CGRP-IR images are merged in **(C)** to show the extent of double labeling in simple, but not ramifying nerve terminals. Colored bars in the merged orthogonal projection indicate the approximate locations of the basal epithelium (green), wing cell layers (blue) and the squamous cell layer (red). Scale bars = 50 μm.

**Figure 8:**
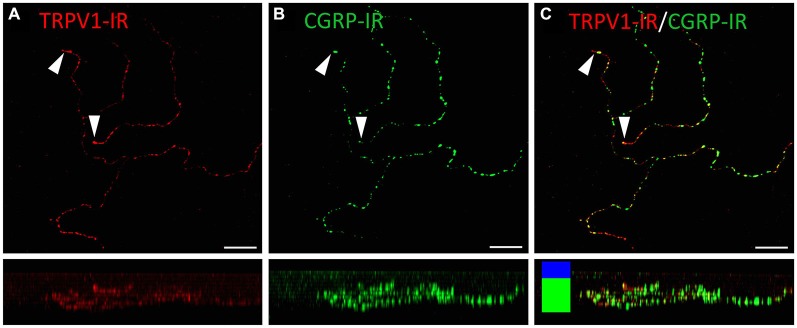
**Confocal images of TRPV1-IR (A) and CGRP-IR (B) nerve terminals in the same field of view restricted to deep in the corneal epithelium**. Images are projected from *z*-series through the basal layer of the corneal epithelium. The small panels under each image show orthogonal views generated by projecting the *z*-series above it in the *x*-plane. Arrowheads point to TRPV1-IR simple nerve terminals that end in the basal epithelium. These were always CGRP-IR. The TRPV1-IR and CGRP-IR images are merged in **(C)** to show the extent of double labeling. Colored bars in the merged orthogonal projection indicate the approximate locations of the basal epithelium (green) and wing cell layers (blue). Scale bars = 30 μm.

**Figure 9:**
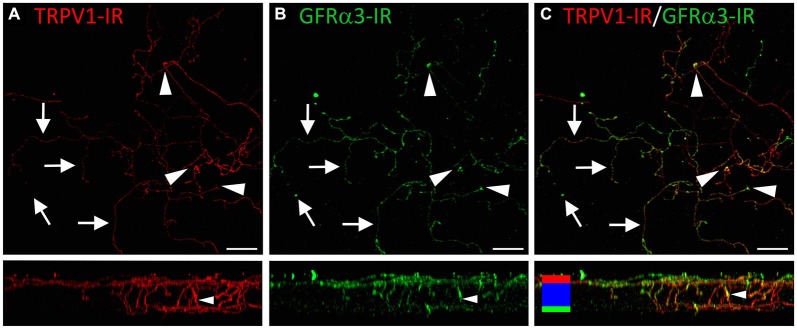
**Confocal images of TRPV1-IR (A) and GFRα3-IR (B) nerve terminals in the same field of view in the corneal epithelium**. Images are projected from *z*-series through the entire corneal epithelium. The small panels under each image show orthogonal views generated by projecting the *z*-series above it in the *x*-plane. Arrows point to TRPV1-IR ramifying nerve terminals in the squamous cell layer and arrowheads point to simple endings that are both TRPV1 and GFRα3-IR. The TRPV1-IR and GFRα3-IR images are merged in **(C)** to show the extent of double labeling in both simple and ramifying nerve terminals. Colored bars in the merged orthogonal projection indicate the approximate locations of the basal epithelium (green), wing cell layers (blue) and the squamous cell layer (red). Scale bars = 30 μm****.

TRPV1-IR nerve terminals were identified throughout the corneal epithelium (Figure 6B). The majority extended from the leash bundles in the basal epithelium towards the surface of epithelium, and terminated as either simple endings in the wing cell layers, or as ramifying endings in the most superficial (squamous) layer of the corneal epithelium (Figure 6B). However, some ended as simple endings in the basal epithelium. TRPV1-IR nerve terminals were never complex. Double labeling of TRPV1-IR endings with antibodies directed against CGRP revealed that CGRP-IR is confined to the simple TRPV1-IR nerve terminals in the wing cell layers (Figure 7) and the basal epithelium (Figure 8). In contrast, GFRα3-IR occurred in both the simple TRPV1-IR nerve terminals in the wing cell layers and the ramifying TRPV1-IR nerve terminals in the most superficial layer of the epithelium (Figure 9), but not in the simple terminals in the basal epithelium. Double labeling of nerve terminals with antibodies directed against CGRP and GFRα3 confirmed that simple endings in the wing cell layers express both CGRP and GFRα3, ramifying endings at the surface express only GFRα3, and simple endings in the basal epithelium expressed CGRP but not GFRα3 (data not shown).

## Discussion

The present study demonstrates that a substantial proportion of corneal afferent neurons (approximately 45%) express TRPV1. The majority of these neurons are likely to be polymodal nociceptors because they were distinct from the corneal pure mechano-nociceptors (expressing Piezo2) and only a very small proportion of TRPV1-IR neurons expressed the cold sensing ion channel TRPM8. The findings also demonstrate that at least three subpopulations of TRPV1-IR neurons innervate the corneal epithelium, which can be distinguished on the basis of molecular phenotype and nerve terminal morphology.

### Corneal TRPV1-IR Neurons are Predominately Polymodal Nociceptors

We have previously identified retrograde labeled corneal afferent neurons on the basis of their molecular phenotype (Ivanusic et al., 2013; Bron et al., 2014). TRPM8 is a non-selective, calcium-permeable, cation channel that is activated by cooling and menthol and is important for cold sensation (McKemy et al., 2002; Peier et al., 2002; Parra et al., 2010), including cold pain (Knowlton et al., 2013). Thus we used TRPM8-IR to identify cold-sensing corneal afferent neurons. Piezo2 is a protein that generates robust mechanically sensitive currents in a number of cultured cell lines (Coste et al., 2010) and RNA interference knockdown of this protein suppresses mechanically activated currents in rapidly adapting DRG neurons (Coste et al., 2010). Thus we used Piezo2 mRNA expression to identify putative corneal pure mechano-nociceptors (Bron et al., 2014). In the present study, TRPV1-IR corneal afferent neurons did not express Piezo2 and only ~6% of these neurons co-expressed TRPM8. The population of neurons that co-expressed TRPV1 and TRPM8 (~31% of TRPM8 neurons) was not further investigated but it is possible that they represent a subpopulation of cold-sensing neurons that display a paradoxical response to noxious heating (Hirata et al., 2012). Given that corneal polymodal nociceptors respond to chemical stimuli, temperature changes (heating) and mechanical stimulation (Belmonte et al., 1991; Gallar et al., 1993), that the TRPV1 channel responds to many of these same stimuli (Caterina et al., 1997; Tominaga et al., 1998; Davis et al., 2000), and that we have shown that TRPV1-IR is not a feature of putative pure mechano-nociceptors, our data strongly suggests that the majority (>90%) of TRPV1-IR corneal afferent neurons must be polymodal nociceptors.

TRPV1-IR neurons were mostly small and unmyelinated (not NF200-IR) cells. These findings are consistent with other reports of TRPV1-IR neurons in both DRG and TG (Orozco et al., 2001; Bae et al., 2004; Murata and Masuko, 2006; Nakamura et al., 2007) and in sensory neurons innervating other specific tissue types; for example rat dura (McIlvried et al., 2010), dental pulp (Gibbs et al., 2011), tongue (Kanazawa and Matsumoto, 2014) and trachea (Yamamoto et al., 2007), and mouse gut (Malin et al., 2009; Tan et al., 2009) and bladder (Nandigama et al., 2013). In addition, our finding that TRPV1 neurons form ~45% of the corneal sensory neurons is consistent with electrophysiological reports that polymodal nociceptors are the most common of the three main classes of corneal sensory neurons (Belmonte and Gallar, 1996). Using TRPV1-IR, TRPM8, and Piezo2 mRNA expression we can account for ~80% of corneal afferent neurons. As we studied constitutive expression of the molecules in the current study, it is possible that some members of the three functional subpopulations of corneal afferent neurons may not express their respective molecular marker under normal conditions, but switch on *de novo* expression in pathological conditions (such as inflammation). An example of such could include silent nociceptors, that under normal conditions are not activated by any stimuli at all, but after inflammation, become sensitive to one or more stimulus types (Michaelis et al., 1996). Alternatively, it is possible that unlabeled neurons represent another as yet undefined subpopulation of corneal afferent neurons, or even polymodal nociceptors that do not express TRPV1. An example of the latter has been reported in mice, where MAS-related G-protein coupled receptor, member D (MrgprD) expression defines a population of non-peptidergic cutaneous polymodal nociceptors that do not express TRPV1 (Zylka et al., 2005; Rau et al., 2009).

### TRPV1 Expressing Corneal Afferent Neurons can be Distinguished on the Basis of their Nerve Terminal Morphology and Molecular Phenotype

**Figure 10:**
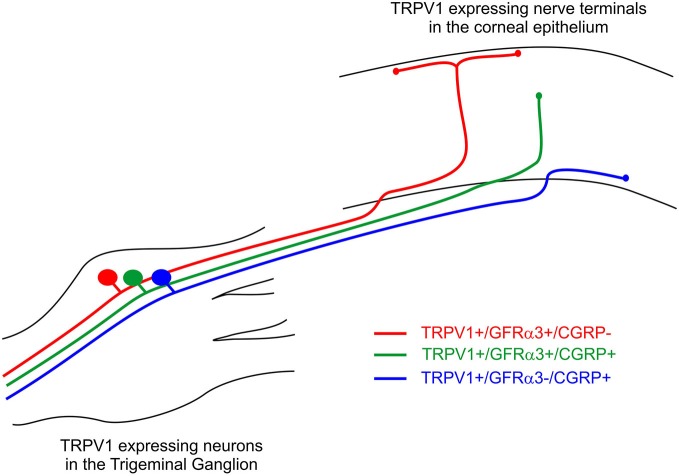
**Schematic of the three different subpopulations of TRPV1 expressing corneal afferent neurons we reported in the trigeminal ganglion and corneal epithelium**.

In a previous study, we have defined the morphology of corneal afferent nerve terminals on the basis of their branching pattern within the epithelium, as either simple, ramifying or complex (Ivanusic et al., 2013). In the present study, we could define two subpopulations of TRPV1-IR nerve terminal endings that approach the surface of the corneal epithelium on the basis of their morphology and molecular phenotype. Simple TRPV1-IR endings in the wing cell layers expressed both CGRP and GFRα3, whilst the more superficially located ramifying TRPV1-IR endings expressed only GFRα3. We could also define a third population of TRPV1-IR nerve terminals that were CGRP-IR but not GFRα3-IR, and terminated as simple endings in the basal epithelium. Together these findings provide clear evidence that TRPV1-IR nerve terminals in the corneal epithelium can be split into separate subpopulations based on their nerve terminal morphology and molecular phenotype (Figure 10).

The molecular phenotypes of the TRPV1-IR nerve endings reported match that of retrograde labeled corneal afferent neurons. The finding that there were many TRPV1-IR corneal afferent neurons that expressed GFRα3, taken together with the findings that some GFRα3-IR neurons expressed CGRP, and some did not, suggests that there must be at least two populations of TRPV1-IR corneal afferent neurons; one that expresses GFRα3 and CGRP and one that expresses GFRα3 but not CGRP. It is unlikely that any of the GFRα3-IR neurons contribute to a significant separate population of neurons that do not express TRPV1 because almost all GFRα3-IR neurons were TRPV1-IR (Table 4). In addition, our finding that there were many CGRP-IR corneal afferent neurons that did not express GFRα3, taken together with the finding that almost all CGRP-IR corneal afferent neurons express TRPV1, suggests that there is an additional population of TRPV1-IR corneal afferent neurons that express CGRP but not GFRα3.

Our findings are consistent with previous studies that have reported co-expression of TRPV1, GFRα3 and/or CGRP in DRG or TG neurons. Almost all GFRα3 neurons in the mouse and rat DRG express TRPV1 (Orozco et al., 2001; Malin et al., 2006) and are small, peripherin-IR (unmyelinated) neurons (Orozco et al., 2001). Many of the GFRα3 neurons reported in these studies express CGRP but some do not (Orozco et al., 2001). In mice, the majority (65%) of TRPV1-IR DRG neurons are GFRα3-IR, and artemin (the ligand for GFRα3) significantly potentiates capsaicin-evoked calcium currents in the same proportion of TRPV1-IR neurons (Malin et al., 2006). CGRP-IR is also observed in over half of rat TRPV1-IR TG neurons (Bae et al., 2004; Murata and Masuko, 2006). GFRα3-IR afferent neurons that innervate the rat dura are both TRPV1-IR and CGRP-IR (McIlvried et al., 2010). About 40% of TRPV1-IR dural afferents are GFRα3-IR, and 20% of dural afferents contain both CGRP and GFRα3 (McIlvried et al., 2010). Whilst there appears to be differences in the proportion of TRPV1 neurons that contain either CGRP or GFRα3 between studies (perhaps species specific), it is clear that these markers potentially identify different subpopulations across a number of species. The current study extends these findings to show that different combinations of these markers can be used to identify specific subpopulations of TRPV1-IR corneal afferent neurons in the guinea pig.

GFRα3 expression has been reported in tyrosine hydroxylase-IR sympathetic postganglionic neurons and their nerve fibers innervating the vasculature of the dura (McIlvried et al., 2010). However, corneal GFRα3-IR nerve terminals are unlikely to be sympathetic or TH-IR, because we have previously shown TH-IR nerve fibers are only rarely found in the basal epithelium of the guinea pig cornea, and are not present in the more superficial layers of the epithelium where we have noted GFRa3-IR nerve terminal endings in the present study (Ivanusic et al., 2013).

### Polymodal Nociceptors with Different Functional Properties have Different Nerve Terminal Morphologies and Epithelial Distribution

Our results suggest that multiple subpopulations of corneal polymodal nociceptors exist, and that these subpopulations have different molecular phenotypes, nerve terminal morphologies and epithelial distributions within the cornea. This is consistent with findings from electrophysiological studies suggesting differences in epithelial distribution of polymodal nociceptors with different functional properties. In both cat (Belmonte et al., 1991) and rabbit (MacIver and Tanelian, 1993), polymodal units that have mechanical and thermal, but not chemical, sensitivity have been reported to conduct predominantly in the Aδ range, whilst polymodal units that displayed responses to all of these stimuli conduct in the C fiber range. Because the C-type nociceptors have higher mechanical thresholds, it has been speculated that their sensory terminals might be located more deeply in the cornea. We speculate further that responses generated by the C-type polymodal nociceptors in these previous studies arise from the simple endings we have reported in the wing cell layers or even in the basal epithelium in our study.

There is evidence of morphologically distinct patterns of innervation for polymodal nociceptors in skin. Cutaneous polymodal receptors are often divided into two largely distinct groups, peptidergic and non-peptidergic, on the basis of whether or not they express the neuropeptides CGRP and/or substance P. In mice, cutaneous non-peptidergic neurons express MrgprD and in glabrous skin these form terminals in the epidermis that are morphologically discrete from those that express CGRP and TRPV1 (Zylka et al., 2005). Terminals of the MrgprD+ fibers appeared to branch as they ascended through the epidermis, and terminated in the stratum granulosum. In contrast, terminals of CGRP+ fibers terminated lower in the epidermis (in the stratum spinosum) as simple endings like those reported in our study. Both MrgprD+ and CGRP+ neurons supplying glabrous skin have been shown to be polymodal nociceptors (Rau et al., 2009). However, selective ablation of the MrgprD+ neurons indicates that they contribute to behavioral responses associated with noxious mechanical stimuli, but not to noxious heat, whilst selective block of signaling by TRPV1 expressing peptidergic neurons impairs the behavioral responses to noxious heat stimuli (Cavanaugh et al., 2009).

### Functions of Corneal TRPV1 Polymodal Nociceptors Expressing GFRa3 and/or CGRP

CGRP has an established role in maintaining corneal integrity via initiating neurogenic corneal inflammation and in promoting wound healing following corneal damage. It acts at receptors on corneal epithelial cells to stimulate synthesis of interleukin-8, which is a chemo-attractant for neutrophils that play an important role in corneal inflammation (Tran et al., 2000a,b). On its own CGRP has no effect on epithelial cell proliferation, but at a low concentration (25 nM) it is reported to act synergistically with substance P to stimulate growth of corneal epithelial cells (Reid et al., 1993) whereas at a higher concentration (1 μm) it inhibits substance P-induced proliferation of corneal epithelial cells (Garcia-Hirschfeld et al., 1994). Application of capsaicin to the cornea promotes the release of CGRP, whereas this effect was not observed when cold stimuli were applied, consistent with release from polymodal nociceptors (Belmonte et al., 2003). Thus the CGRP-IR and TRPV1-IR corneal afferent nerve fibers we have reported to terminate in basal and wing cell layers of the epithelium in the present study are likely to have an important role maintaining corneal integrity.

In contrast to CGRP, there is no information about how artemin/GFRα3 signaling affects corneal function. There is increasing evidence that artemin/GFRα3 signaling is involved in peripheral sensitization in DRG neurons, and that the TRPV1 ion channel is involved in this process (Malin and Davis, 2008). Artemin expression increases in experimentally-induced inflammation of the footpad (Malin et al., 2006). Furthermore, TRPV1 expression is increased in sensory neurons in animals overexpressing artemin (Elitt et al., 2006, 2008) or after artemin application to the footpad (Malin et al., 2006), and this may contribute to thermal hyperalgesia following inflammation. Artemin also potentiates responses of TRPV1 channels to capsaicin *in vitro* (Malin et al., 2006). We suggest that the GFRα3 expressing subpopulations of corneal polymodal nociceptors demonstrated in the present study can be sensitized by artemin and that artemin/GFRα3 signaling may therefore be involved in modulating sensitivity of at least some corneal polymodal nociceptors.

In conclusion, this study demonstrates that TRPV1 expressing corneal polymodal sensory neurons can be subdivided into at least three subpopulations on basis of their molecular phenotype and nerve terminal morphology. The functional significance of these subpopulations has not been resolved, but it seems likely that each of them will be tuned both molecularly and structurally to respond preferentially to particular types of stimuli. The demonstration of GFRα3 in many of the TRPV1 expressing corneal neurons uncovers a potential novel role for artemin/GFRα3 signaling in regulating corneal sensation.

## Author and Contributors

JI and JB contributed to the conception and design of the work and AA, RB, and JI contributed to the acquisition, analysis, and interpretation of data. All authors contributed to drafting and revising the manuscript, and approved the final submitted version. All authors also agree to be accountable for all aspects of the work and ensure that questions related to the accuracy or integrity of any part of the work will be appropriately investigated and resolved.

## Conflict of Interest Statement

The authors declare that the research was conducted in the absence of any commercial or financial relationships that could be construed as a potential conflict of interest.

## Abbreviations

CGRP: Calcitonin gene-related peptide
DRG: Dorsal root ganglion
GFRα3: Glial cell line-derived neurotrophic factor family receptor alpha3
IR: Immunoreactive
MrgprD: MAS-related G-protein coupled receptor, member D
TG: Trigeminal ganglion
TRPM8: Transient receptor potential cation channel subfamily M member 8
TRPV1: Transient receptor potential cation channel subfamily V member 1.
